# Selective serotonin reuptake inhibitor use in hip fracture patients: a Danish nationwide prevalence study

**DOI:** 10.1080/17453674.2018.1543842

**Published:** 2018-12-10

**Authors:** Stine B Bruun, Irene Petersen, Nickolaj R Kristensen, Deirdre Cronin-Fenton, Alma B Pedersen

**Affiliations:** a Department of Clinical Epidemiology, Aarhus University Hospital, Aarhus, Denmark;; b Department of Primary Care and Population Health, University College London, UK

## Abstract

Background and purpose — Selective serotonin reuptake inhibitors (SSRIs) are often used in the elderly and are associated with adverse effects. Therefore, it is important to ascertain the prevalence of SSRI use in fragile and surgery-treated hip fracture patients.

Methods — This population-based prevalence study included Danish hip fracture patients aged ≥ 65 years operated in 2006–2016 (n = 68,607) and matched individuals from the background population (n = 343,020). Using Poisson regression, prevalence risk ratios (PRRs) with 95% confidence intervals (CIs) were estimated to compare hip fracture patients with the general population, and to estimate the association between hip fracture patient characteristics and SSRI prescriptions.

Results — The prevalence of SSRI use among hip fracture patients was 23% compared with 12% in the general population. During 2006–2016, the prevalence decreased from 26% to 18% among hip fracture patients and from 13% to 10% in the general population. Factors associated with SSRI use in hip fracture patients were age 75–84 years (PRR 1.18, CI 1.13–1.23), age ≥ 85 years (PRR 1.17, CI 1.11–1.22), female sex (PRR 1.13, CI 1.09–1.17), unmarried status (PRR 1.15, CI 1.11–1.19), living in a residential institution (PRR 2.30, CI 2.19–2.40), Charlson comorbidity index (CCI) score 1–2 (PRR 1.50, CI 1.45–1.55), CCI score 3+ (PRR 1.62, CI 1.55–1.69), and several medications.

Interpretation — The prevalence of SSRI use was high among hip fracture patients compared with the general population. Our data stress the importance of continued clinical awareness of frailty, comorbidity, and polypharmacy of hip fracture patients and the potentially adverse drug effects of SSRI treatment.

Hip fracture affects approximately 6,500 elderly aged 65 years or older every year in Denmark (5.7 million inhabitants) (Centre for Clinical Epidemiology and Biostatistics North 2017, Statistics Denmark 2017). Most hip fractures in the elderly result from falls; only 5% of hip fracture patients have no fall history (Parker and Johansen 2006). Falls in the elderly are associated with several risk factors including gait and balance impairment, frailty, disability, comorbid conditions, and polypharmacy (Vieira et al. 2016). Polypharmacy is frequent in the elderly and often includes use of anticoagulants, statins, antipsychotics, and antidepressants (Maher et al. 2014). The most commonly prescribed antidepressant drugs are selective serotonin reuptake inhibitors (SSRIs) (Mars et al. 2017). SSRI use is associated with an increased risk of falls resulting in an emergency department visit or hospitalization (Macri et al. 2017). SSRI use is further associated with an increased risk of hip fracture (Souverein et al. 2016), which may be due to bone loss, hyponatremia, or hemodynamic disturbances (Bakken et al. 2013).

SSRI use may be more prevalent in hip fracture patients compared with the general population (Hung et al. 2017). However, there are a limited number of studies on this. Furthermore, factors associated with SSRI use in hip fracture patients are not identified. One study in individuals aged 65 years or older without hip fracture found that SSRI users were more likely to be women and aged 75–94 years (Marengoni et al. 2016). SSRI use was strongly associated with use of other drugs such as other psychiatric drugs, statins, antihypertensives, and anticoagulants (Marengoni et al. 2016). Another study found an association between hypertension, dementia, depression, and polypharmacy and potentially inappropriate medication use including SSRI use in individuals aged 65 years or older receiving health care services (Alhmoud et al. 2015). Some of these associated factors may contribute to the increased risk of hip fracture in SSRI users. However, no previous studies have investigated this.

We therefore assessed the prevalence of SSRI use in elderly hip fracture patients before the fracture compared with the prevalence of SSRI use in the general population. Furthermore, we aimed to identify factors associated with SSRI use in hip fracture patients before occurrence of the fracture. 

## Methods

### Setting and design

The study is a nationwide cohort study using prospectively collected data from Danish medical registries. The health care system in Denmark is tax-funded, and all 5.7 million citizens have equal access to health care services (Statistics Denmark 2017).

### Data sources

The Danish Multidisciplinary Hip Fracture Registry (Sørensen et al. 2009) contains detailed pre- and postoperative clinical data on all patients aged 65 years or older with first-time hip fracture admitted to any orthopedic department in Denmark since 2004. These data were linked to data from the Danish Civil Registration System (Schmidt et al. 2014). The Danish Civil Registration System was established in 1968 and holds data on date of birth, vital status, and migration on all individuals in Denmark. Every citizen has a unique civil personal registration number, which allows for individual-level linkage across all Danish registries. First, data were linked to the Danish National Patient Registry (Schmidt et al. 2015), which contains data on civil personal registration number, hospital admission and discharge diagnosis codes, and diagnostic and surgical procedure codes from all Danish somatic hospitals since 1977. Diagnoses were coded using the International Classification of Diseases Eighth Revision (ICD-8) until the end of 1993 and Tenth Revision (ICD-10) thereafter. Furthermore, data were linked to the Danish National Database of Reimbursed Prescriptions (Johannesdottir et al. 2012), which tracks reimbursed medicine dispensing at all community pharmacies and hospital-based outpatient pharmacies in Denmark since 2004. Prescription information on individuals living in nursing homes is included in the database as they receive medication from community pharmacies. The database holds data on civil personal registration number, Anatomical Therapeutic Chemical code, redemption date, item quantity, pack size, defined daily dose, dose form, and generic substitution done at pharmacy.

### Study population

Hip fracture patients aged 65 years or older undergoing surgical treatment for hip fracture between 1 January 2006 and 31 December 2016 were identified in the Danish Multidisciplinary Hip Fracture Registry. Using the Civil Registration System, each hip fracture patient was frequency matched with up to 5 individuals from the general population on age and sex at the time of surgery (index date).

### Variables

Information on age and sex of all participants was obtained from the Danish Civil Registration System. 3 age categories were created: 65–74 years, 75–84 years, and ≥ 85 years. Comorbidities of all participants were identified using the Danish National Patient Registry. Overall comorbidity was summarized according to the Charlson Comorbidity Index (CCI) score. The CCI was categorized as low (score 0), medium (score 1–2), and high (score ≥ 3) comorbidity score. The following medications were assessed from the Danish National Database of Reimbursed Prescriptions: SSRIs, non-steroid anti-inflammatory drugs (NSAIDs), corticosteroids, anticoagulants, statins, non-SSRI antidepressants, and antipsychotics. Use of each medication including SSRIs was defined as follows: users redeemed at least 1 prescription within 1 year and non-users redeemed no prescriptions within 365 days before hip fracture surgery date (index date). Marital status of hip fracture patients was obtained from the Danish Civil Registration System. Housing, operation year, and BMI information of hip fracture patients were assessed from the Danish Multidisciplinary Hip Fracture Registry. 4 categories comprising housing information were created: own accommodation, residential institution, homeless, and unknown. Likewise, 5 categories based on BMI values were created: underweight (BMI < 18.5), normal weight (BMI ≥ 18.5 < 25), overweight (BMI ≥ 25 < 30), obese (BMI ≥ 30), and unknown. All codes defining study variables are available in Tables 1–3 in the Supplementary data.

**Table 4.** Participant characteristics according to selective serotonin reuptake inhibitor (SSRI) use 2006–2016. Values are frequency (%)

*Variable*	*All participants*	*Hip fracture patients*		*General population*	
		*SSRI users*	*Non-users*	*SSRI users*	*Non-users*
*Total*	*411,627 (100)*	*16,081 (23)*	*52,526 (77)*	*41,191 (12)*	*301,829 (88)*
*Median age*	*83 (64–108)*	*84 (65–105)*	*83 (65–108)*	*86 (64–106)*	*83 (64–108)*
*Age*					
* 65–74*	*79,667 (19)*	*2,705 (20)*	*10,585 (80)*	*4,690 (7)*	*61,687 (93)*
* 75–84*	*157,030 (38)*	*6,331 (24)*	*19,797 (76)*	*14,569 (11)*	*116,333 (89)*
* *≥ *85*	*174,930 (43)*	*7,045 (24)*	*22,144 (76)*	*21,932 (15)*	*123,809 (85)*
*Sex*					
* Male*	*118,706 (29)*	*4,219 (21)*	*15,567 (79)*	*8,288 (8)*	*90,632 (92)*
* Female*	*292,921 (71)*	*11,862 (24)*	*36,959 (76)*	*32,903 (13)*	*211,197 (87)*
*Operation year*					
* 2006*	*37,681 (9)*	*1,618 (26)*	*4,663 (74)*	*4,070 (13)*	*27,330 (87)*
* 2007*	*38,406 (9)*	*1,687 (26)*	*4,714 (74)*	*4,102 (13)*	*27,903 (87)*
* 2008*	*41,028 (10)*	*1,753 (26)*	*5,085 (74)*	*4,370 (13)*	*29,820 (87)*
* 2009*	*37,351 (9)*	*1,599 (26)*	*4,627 (74)*	*3,979 (13)*	*27,146 (87)*
* 2010*	*39,024 (10)*	*1,684 (26)*	*4,820 (74)*	*4,183 (13)*	*28,337 (87)*
* 2011*	*39,012 (9)*	*1,591 (24)*	*4,911 (76)*	*4,163 (13)*	*28,347 (87)*
* 2012*	*37,230 (9)*	*1,442 (23)*	*4,763 (77)*	*3,815 (12)*	*27,210 (88)*
* 2013*	*37,255 (9)*	*1,320 (21)*	*4,890 (79)*	*3,583 (12)*	*27,462 (88)*
* 2014*	*36,462 (9)*	*1,209 (20)*	*4,868 (80)*	*3,203 (11)*	*27,182 (89)*
* 2015*	*36,180 (9)*	*1,206 (20)*	*4,824 (80)*	*3,118 (10)*	*27,032 (90)*
* 2016*	*31,998 (8)*	*972 (18)*	*4,361 (82)*	*2,605 (10)*	*24,060 (90)*
*CCI score*					
* Low (0)*	*255,954 (62)*	*6,470 (19)*	*27,979 (81)*	*20,459 (9)*	*201,046 (91)*
* Medium (1–2)*	*121,213 (30)*	*6,917 (28)*	*17,999 (72)*	*15,894 (17)*	*80,403 (83)*
* High (3+)*	*34,460 (8)*	*2,694 (29)*	*6,548 (71)*	*4,838 (19)*	*20,380 (81)*
*Other medication*					
* NSAIDs*	*85,307 (21)*	*3,559 (24)*	*11,033 (76)*	*8,677 (12)*	*62,038 (88)*
* Corticosteroids*	*34,400 (8)*	*1,884 (27)*	*5,214 (73)*	*4,224 (15)*	*23,078 (85)*
* Anticoagulants*	*184,999 (45)*	*9,077 (27)*	*24,463 (73)*	*22,373 (15)*	*129,086 (85)*
* Statins*	*111,799 (27)*	*4,565 (26)*	*13,040 (74)*	*11,711 (12)*	*82,483 (88)*
* Non-SSRI anti-depressants*	*39,550 (10)*	*3,798 (38)*	*6,117 (62)*	*9,683 (33)*	*19,952 (67)*
* Antipsychotics*	*20,579 (6)*	*2,683 (42)*	*3,669 (58)*	*5,123 (36)*	*9,104 (64)*

CCI: Charlson Comorbidity Index

**Table 6.** Selective serotonin reuptake inhibitor (SSRI) use in hip fracture patients compared with SSRI use in the general population sample

*Variable*	*Unadjusted PRR (95% CI)*	*Adjusted PRR*^a^*(95% CI)*
*Total*	*2.0 (1.9–2.0)*	*2.0 (1.9–2.0)*
*Age*		
* 65–74*	*2.9 (2.8–3.0)*	*2.9 (2.8–3.0)*
* 75–84*	*2.2 (2.1–2.2)*	*2.2 (2.1–2.2)*
* ≥ 85*	*1.6 (1.6–1.7)*	*1.6 (1.6–1.7)*
*Sex*		
* Male*	*2.5 (2.5–2.6)*	*2.5 (2.5–2.6)*
* Female*	*1.8 (1.8–1.8)*	*1.8 (1.8–1.8)*
*CCI score*		
* Low (0)*	*2.0 (2.0–2.1)*	*1.9 (1.9–2.0)*
* Medium (1–2)*	*1.7 (1.6–1.7)*	*1.7 (1.7–1.8)*
* High (3+)*	*1.5 (1.5–1.6)*	*1.5 (1.5–1.6)*
*Other medication*		
* NSAIDs*	*2.0 (1.9–2.1)*	*2.0 (1.9–2.1)*
* Corticosteroids*	*1.7 (1.6–1.8)*	*1.8 (1.7–1.9)*
* Anticoagulants*	*1.8 (1.8–1.9)*	*1.9 (1.8–1.9)*
* Statins*	*2.1 (2.0–2.2)*	*2.1 (2.0–2.2)*
* Non-SSRI antidepressants*	*1.2 (1.1–1.2)*	*1.2 (1.2–1.3)*
* Antipsychotics*	*1.2 (1.1–1.2)*	*1.2 (1.1–1.3)*

**^a^***Adjusted for age and sex.*

CCI: Charlson Comorbidity Index

**Table 7.** Prevalence risk ratios (PRRs) of selective serotonin reuptake inhibitor (SSRI) use according to hip fracture patients’ characteristics

*Variable*	*Unadjusted PRR(95% CI)*	*Adjusted PRR*^a^*(95% CI)*
*Age*		
* 65–74*	*1.0*	*1.0*
* 75–84*	*1.2 (1.1–1.3)*	*1.2 (1.1–1.2)*
* *≥ *85*	*1.2 (1.1–1.2)*	*1.2 (1.1–1.2)*
*Sex*		
* Male*	*1.0*	*1.0*
* Female*	*1.1 (1.1–1.2)*	*1.1 (1.1–1.2)*
*Marital status*		
* Married*	*1.0*	*1.0*
* Unmarried*	*1.2 (1.2–1.2)*	*1.2 (1.1–1.2)*
*Housing*		
* Own accommodation*	*1.0*	*1.0*
* Homeless*	*0.5 (0.1–2.0)*	*0.5 (0.1–2.1)*
* Residential institution*	*2.3 (2.2–2.4)*	*2.3 (2.2–2.4)*
*Operation year*		
* 2006*	*1.0*	*1.0*
* 2007*	*1.0 (1.0–1.1)*	*1.0 (1.0–1.1)*
* 2008*	*1.0 (0.9–1.1)*	*1.0 (0.9–1.1)*
* 2009*	*1.0 (0.9–1.1)*	*1.0 (0.9–1.1)*
* 2010*	*1.0 (0.9–1.1)*	*1.0 (0.9–1.1)*
* 2011*	*1.0 (0.9–1.0)*	*1.0 (0.9–1.0)*
* 2012*	*0.9 (0.8–1.0)*	*0.9 (0.8–1.0)*
* 2013*	*0.8 (0.8–0.9)*	*0.8 (0.8–0.9)*
* 2014*	*0.8 (0.7–0.8)*	*0.8 (0.7–0.8)*
* 2015*	*0.8 (0.7–0.8)*	*0.8 (0.7–0.8)*
* 2016*	*0.7 (0.7–0.8)*	*0.7 (0.7–0.8)*
*Body mass index*		
* < 18.5: Underweight*	*1.1 (1.0–1.1)*	*1.0 (1.0–1.1)*
* *≥ *18.5 < 25: Normal weight*	*1.0*	*1.0*
* *≥ *25: Overweight or obese*	*1.0 (1.0–1.0)*	*1.0 (1.0–1.1)*
*Charlson comorbidity index*		
* Low (0)*	*1.0*	*1.0*
* Medium (1–2)*	*1.5 (1.4–1.5)*	*1.5 (1.5–1.6)*
* High (3+)*	*1.6 (1.5–1.6)*	*1.6 (1.6–1.7)*
*Comorbidity*		
* Myocardial infarction*	*1.1 (1.0–1.2)*	*1.1 (1.0–1.2)*
* Congestive heart failure*	*1.1 (1 .1–1.2)*	*1.1 (1.1–1.2)*
* Peripheral vascular disease*	*1.1 (1.1–1.2)*	*1.2 (1.1–1.2)*
* Cerebrovascular disease*	*1.6 (1.5–1.6)*	*1.6 (1.5–1.7)*
* Dementia*	*2.0 (1.9–2.1)*	*2.0 (1.9–2.1)*
* Chronic pulmonary disease*	*1.3 (1.2–1.3)*	*1.3 (1.2–1.3)*
* Connective tissue disease*	*1.0 (0.9–1.1)*	*1.0 (0.9–1.1)*
* Ulcer disease*	*1.3 (1.3–1.4)*	*1.3 (1.3–1.4)*
* Liver disease*	*1.1 (1.0–1.3)*	*1.2 (1.0–1.3)*
* Diabetes type 1 and 2*	*1.1 (1.0–1.1)*	*1.1 (1.1–1.2)*
* Hemiplegia*	*1.6 (1.2–2.0)*	*1.6 (1.3–2.1)*
* Moderate to severe renal disease*	*1.1 (1.1–1.2)*	*1.2 (1.1–1.3)*
* Cancer*	*1.0 (1.0–1.1)*	*1.0 (1.0–1.1)*
*Other medication*		
* NSAIDs*	*1.1 (1.0–1.1)*	*1.1 (1.0–1.1)*
* Corticosteroids*	*1.2 (1.1–1.2)*	*1.2 (1.1–1.2)*
* Anticoagulants*	*1.4 (1.3–1.4)*	*1.4 (1.3–1.4)*
* Statins*	*1.2 (1.1–1.2)*	*1.2 (1.1–1.2)*
* Non-SSRI antidepressants*	*1.8 (1.8–1.9)*	*1.8 (1.8–1.9)*
* Antipsychotics*	*2.0 (1.9–2.0)*	*1.0 (1.9–2.1)*

**^a^***Adjusted for age and sex.*

### Statistics

The annual prevalence of SSRI prescription redemption both overall and stratified by generic type was calculated in both hip fracture patients and the general population sample. SSRI use in hip fracture patients was compared with SSRI use in the general population sample using Poisson regression analyses and stratifying by age, sex, CCI, and other medication use. The model was evaluated for effect modification by age and sex. Crude and adjusted prevalence risk ratios (PRR) of SSRI use in hip fracture patients were estimated according to patient characteristics using Poisson regression analysis for comparison. The regression analyses were adjusted for age and sex. All statistical analyses were performed using Stata 14 for Windows (Stata Corp, College Station, TX, USA).

### Ethics, funding, and potential conflicts of interest

Ethical approval was not required. As this study did not involve contact with patients or an intervention, it was not necessary to obtain permission from the Danish Scientific Ethical Committee. The study was approved by the Danish Data Protection Agency (record number: 1-16-02-467-15). This work was supported by the Independent Research Fund Denmark (grant number 6120-00034). There are no conflicts of interest to declare. 

## Results

We identified 68,607 first-time hip fracture patients aged 65 years or older operated between 2006 and 2016 and 343,020 age- and sex-matched individuals from the general population. 16,081 (23%) hip fracture patients were SSRI users compared with 41,191 (12%) in the general population sample. Most participants were women (71%) with a median age of 83 (64–108) years at the index date (Table 4). Overall, 62% of the participants had a low CCI score, 29% had a medium CCI score, and 8% had a high CCI score, but the proportion of SSRI users increased with increasing CCI score in both hip fracture patients and the general population sample. Use of other medication was common among all participants and NSAIDs, anticoagulants, and statins were prescribed to more than 20% (Table 4). Almost half of the hip fracture patients lived in their own accommodation (31,076, 45%) whereas 7,105 (10%) lived in a residential institution, where the proportion of SSRI users was larger (41%) compared with patients living in their own accommodation (18%). Most hip fracture patients were normal weight (31,635, 46), while 17,640 (26%) were overweight or obese. 30,404 (44%) hip fracture patients were missing housing data and 13,329 (19%) were missing BMI data (Table 5, see Supplementary data).

### Prevalence of SSRI use

Between 2006 and 2016, the overall prevalence of SSRI use decreased from 26% to 18% in hip fracture patients and from 13% to 10% in the general population sample (Table 4 and Figure). Overall, the prevalence of SSRI use in both populations was stable from 2006 to 2011, after which the prevalence decreased until the end of the study period. Citalopram was the most frequently redeemed SSRI and decreased from 18% to 11% in hip fracture patients and from 9% to 6% in the general population sample during the study period. The overall prevalence of SSRI prescriptions decreased except for sertraline prescriptions, which increased from 2% to 4% in hip fracture patients and from 1% to 2% in the general population sample between 2006 and 2016.

Table 6 shows the PRRs of SSRI use in hip fracture patients compared with SSRI use in the general population sample. Overall, hip fracture patients had a higher prevalence of SSRI use compared with the general population. When stratifying by age, hip fracture patients aged 65–74 years had a PRR of 2.88 (CI 2.75–3.02), patients aged 75–84 years had a PRR of 2.18 (CI 2.11–2.24), and patients aged 85 years or older had a PRR of 1.60 (CI 1.56–1.65) compared with the general population of the same age. Male hip fracture patients were more than twice as likely to have SSRI treatment compared with the general population. The prevalence of SSRI treatment for female hip fracture patients was also nearly doubled compared with the general population. Hip fracture patients had a higher prevalence of SSRI use than the general population in all CCI groups and when stratifying on other medication use. However, the PRR was only 1.20 (CI 1.16–1.25) when stratifying on non-SSRI antidepressant use and 1.19 (CI 1.14–1.25) when stratifying on antipsychotic use comparing hip fracture patients with the general population.

**Figure:**
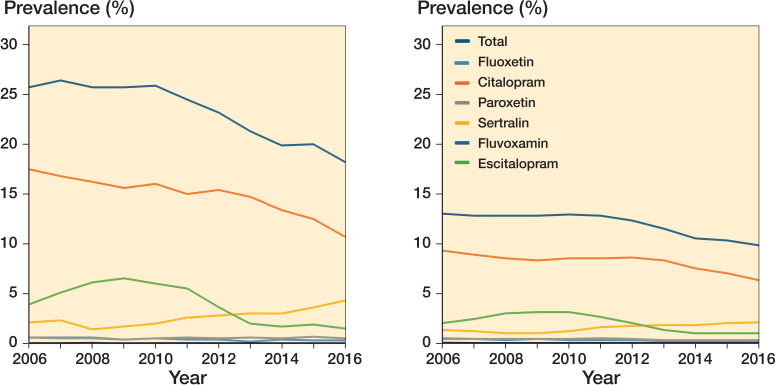
Annual prevalence of selective serotonin reuptake inhibitor use in hip fracture patients (left panel) and in the general population (right panel), 2006–2016, overall and stratified by generic type.

### Factors associated with SSRI use in hip fracture patients

Hip fracture patients aged 75–84 years and 85 years or more were more likely to receive SSRI treatment than hip fracture patients aged 65–74 years. In hip fracture patients, SSRI use was higher among women than among men. Likewise, SSRI use was higher among unmarried hip fracture patients than among married. Living in a residential institution markedly increased the likelihood of receiving SSRI treatment as a hip fracture patient. Medium CCI score and high CCI score in hip fracture patients were also associated with increased use of SSRIs compared with low CCI score. Considering the individual comorbidities, hip fracture patients with dementia were twice as likely to receive SSRI treatment compared with hip fracture patients without dementia. Other comorbidities indicating higher probability of SSRI use among hip fracture patients were myocardial infarction, congestive heart failure, cerebrovascular disease, chronic pulmonary disease, ulcer disease, liver disease, diabetes, hemiplegia, and renal disease. Hip fracture patients using other medication such as NSAIDs, corticosteroids, anticoagulants, and statins had a higher probability of redeeming SSRIs compared with non-users of other medication. Hip fracture patients using non-SSRI antidepressants and antipsychotics were more likely to receive SSRI treatment compared with non-users (Table 7). 

## Discussion

The prevalence of SSRI use among hip fracture patients was approximately double the prevalence in the general population sample during 2006 to 2016, irrespective of age, sex, and CCI score at index date. SSRI prevalence was higher among younger hip fracture patients compared with elderly patients. Citalopram was the most frequently prescribed SSRI in both hip fracture patients and the general population sample. Several factors including age 75 years and above, female sex, unmarried status, living in a residential institution, comorbidity, and use of other medication were associated with higher SSRI use among hip fracture patients.

Strengths of this study include the large nationwide study population, enabling us both to study the use of SSRIs in hip fracture patients and to compare this with use in the general Danish population. The data originate from registries based on a tax-supported and uniformly organized health care system, reducing the risk of recall bias. SSRIs are only available by prescription, which ensures accurate data on the redemption of the medication in the study population. The population of Danish hip fracture patients is similar regarding age and sex to the populations used in other studies on hip fractures in the elderly (Lawrence et al. 2002, Roche et al. 2005). However, the study presents some limitations. First, redemption of the prescribed medication does not necessarily mean that patients were compliant and took the medication. However, prescription redemption is considered a good measure of medication intake even in the presence of misclassification (Schneeweiss and Avorn 2005). Furthermore, patients redeeming 2 prescriptions are more likely to take their medication and a sensitivity analysis in a previous study showed no difference between hip fracture patients redeeming only 1 prescription and those redeeming 2 (Bruun et al. 2018). Second, comorbidity information was obtained from the Danish National Patient Registry, which holds only data on patients treated in the hospital sector. Hence, hip fracture patients may be more likely to have registered comorbidities because they are admitted to hospital. This may lead to either non-differential misclassification or residual confounding. Third, the comorbidity information did not include data on psychiatric diagnoses. However, we included other medication such as NSAIDs, corticosteroids, anticoagulants, statins, non-SSRI antidepressants, and antipsychotics, which may help to estimate the association between psychiatric diagnoses and diagnoses treated in general practice only and SSRI use in hip fracture patients. Fourth, we did not have information on smoking or alcohol use, which may be risk factors for several comorbidities and SSRI use. However, Souverein et al. (2016) found that additional adjustment for these variables had limited impact on effect estimates examining the association between SSRI use and the risk of hip fracture. The CCI included chronic obstructive pulmonary disease, which may function as a surrogate measure of smoking. Fifth, housing and BMI data were incomplete, but previous studies using multiple imputation of BMI on hip fracture data showed no impact on the estimates after adjustment for BMI (Pedersen et al. 2017).

Overall, the prevalence of SSRI use was doubled in hip fracture patients compared with the general population sample. A study conducted in the UK reported a prevalence of SSRI use of 6% in hip fracture patients and 3% in the general population (Hubbard et al. 2003). However, the study was conducted between 1987 and 1999, when SSRIs were generally less used (Mars et al. 2017). Nonetheless, the prevalence ratio between hip fracture patients and the general population is similar to our results. The decrease in prevalence between 2006 and 2016 was reflected in both hip fracture patients and the general population sample. The observed decrease in SSRI prescribing among hip fracture patients aged 65 years and older may be explained by an increased clinical awareness of the adverse effects related to SSRIs, which have been subject to debate during recent years (Topiwala et al. 2014). Our finding that citalopram was the most frequent subtype of prescribed SSRIs is consistent with results obtained from the general UK population (Lockhart and Guthrie 2011, McCrea et al. 2016).

The prevalence of SSRI use increased with increasing age and CCI score, and women had a higher prevalence of SSRI use than men in both hip fracture patients and the general population sample. However, when comparing hip fracture patients with the general population sample the relative prevalence increases with decreasing age and CCI score and is larger in men than in women. This indicates that SSRI use may be more prevalent in male hip fracture patients aged 65–74 years with low CCI score than in female hip fracture patients aged 85 years or older with high CCI score. Bakken et al. (2013) found a more pronounced excess risk of hip fracture among men exposed to SSRIs than among exposed women. This indicates that exposure to SSRIs may affect the risk of hip fracture relatively more in men than in women. Our results support this finding as the relative prevalence of SSRI use in hip fracture patients compared with the general population is higher in men than in women. A Swedish study found that high serum serotonin and SSRI use predicts a lower bone mineral density and an increased risk of hip fracture in elderly men (Kristjansdottir et al. 2018). This may explain the higher proportion of male SSRI users among elderly hip fracture patients compared with the general population. A study by Zhou et al. (2018) further supports this as it shows no difference between female SSRI users and non-users when comparing bone mineral density values. However, further investigations are needed to assess whether the effect of SSRIs is more pronounced in men than in women. Our results further suggest that the younger age group and lower CCI score group of hip fracture patients have a relatively higher prevalence of SSRI use than the older age group and higher CCI score group. Nevertheless, there may be a risk of bias as we only had data on comorbidities treated in the hospital. Hip fracture patients may be more likely to have these comorbidities registered as they were admitted to hospital than the general population sample.

An Italian study investigated factors associated with SSRI use among the elderly aged 65 years or older. The study found an association between SSRI use and female sex, age 75–94 years, and other medication such as psychiatric drugs, statins, and antihypertensives (Marengoni et al. 2016), which is similar to our findings in hip fracture patients. However, we also found an association with unmarried status, living in a residential institution, and several comorbidities. These lifestyle factors and conditions suggests that hip fracture patients receiving SSRI treatment are more fragile than non-users. We expect any potential bias to be non-differential as we do not expect registration of the associated factors to differ between hip fracture patients using SSRIs and non-users. Our results suggest that hip fracture patients using SSRIs may have a more complicated course of treatment than hip fracture patients not treated with SSRIs. Though several factors were associated with SSRI use among elderly hip fracture patients, further studies are needed in order to uncover whether these patients are more prone to complications after surgery.

In summary, this nationwide cohort study based on prospectively collected data from population-based Danish registries found a high prevalence of SSRI use of almost one-quarter among elderly hip fracture patients versus one-tenth in the general population sample. Factors associated with SSRI use in hip fracture patients were age 75 years and above, female sex, unmarried status, living in a residential institution, CCI score 1–2 and ≥ 3, and use of other medication. Our data stress the importance of continued clinical awareness of frailty among hip fracture patients and potentially monitoring any evidence of adverse drug effects of SSRI treatment.

## Supplementary Material

Supplemental Material

## Supplementary data

Supplementary Tables 1–3 and 5 are available in the online version of this article, http://dx.doi.org/10.1080/17453674.2 018.1543842

## Notes

The authors would like to thank the staff of the hospital departments caring for patients with hip fracture for their continuous effort and contribution to acquisition of data in the Danish Multidisciplinary Hip Fracture Registry.

ABP conceived the study. SBB, ABP, DCF, and IP designed the study. SBB and ABP undertook data application. NRK undertook the initial data management with input from SBB, ABP, DCF, and IP. SBB conducted the statistical analyses and drafted the first manuscript, with ABP, DCF, and IP contributing to subsequent iterations. All authors provided critical input on the manuscript. 

*Acta* thanks Jan-Erik Gjertsen and Karl Michaëlsson for help with peer review of this study.

## References

[CIT0001] Alhmoud E, Khalifa S, Bahi AA Prevalence and predictors of potentially inappropriate medications among home care elderly patients in Qatar. Int J Clin Pharm 2015; 37(5): 815–21. 25986290 10.1007/s11096-015-0125-0

[CIT0002] Bakken MS, Engeland A, Engesaeter L B, Ranhoff A H, Hunskaar S, Ruths S Increased risk of hip fracture among older people using antidepressant drugs: data from the Norwegian Prescription Database and the Norwegian Hip Fracture Registry. Age Ageing 2013; 42(4): 514–20. 23438446 10.1093/ageing/aft009

[CIT0003] Bruun S B, Petersen I, Kristensen N R, Cronin-Fenton D, Pedersen A B Selective serotonin reuptake inhibitor use and mortality, postoperative complications, and quality of care in hip fracture patients: a Danish nationwide cohort study. Clin Epidemiol 2018; 10: 1053–71. 30214311 10.2147/CLEP.S166309 PMC6118260

[CIT0004] Centre for Clinical Epidemiology and Biostatistics North The Danish Multidisciplinary Hip Fracture Register—National Annual Report 2017, The Danish Clinical Registries.

[CIT0005] Hubbard R, Farrington P, Smith C, Smeeth L, Tattersfield A Exposure to tricyclic and selective serotonin reuptake inhibitor antidepressants and the risk of hip fracture. Am J Epidemiol 2003; 158(1): 77–84. 12835289 10.1093/aje/kwg114

[CIT0006] Hung S C, Lin C H, Hung H C, Lin C L, Lai S W Use of selective serotonin reuptake inhibitors and risk of hip fracture in the elderly: a case-control study in Taiwan. J Am Med Dir Assoc 2017; 18(4): 350–4. 28159466 10.1016/j.jamda.2016.12.003

[CIT0007] Johannesdottir S A, Horvath-Puho E, Ehrenstein V, Schmidt M, Pedersen L, Sorensen H T Existing data sources for clinical epidemiology: the Danish National Database of Reimbursed Prescriptions. Clin Epidemiol 2012; 4: 303–13. 23204870 10.2147/CLEP.S37587 PMC3508607

[CIT0008] Kristjansdottir H L, Lewerin C, Lerner U H, Waern E, Johansson H, Sundh D, Karlsson M, Cummings S R, Zetterberg H, Lorentzon M, Ohlsson C, Mellstrom D High serum serotonin predicts increased risk for hip fracture and nonvertebral osteoporotic fractures: the MrOS Sweden Study. J Bone Miner Res 2018; 33(9): 1560–7. 29750841 10.1002/jbmr.3443

[CIT0009] Lawrence V A, Hilsenbeck S G, Noveck H, Poses R M, Carson J L Medical complications and outcomes after hip fracture repair. Arch Intern Med 2002; 162(18): 2053–7. 12374513 10.1001/archinte.162.18.2053

[CIT0010] Lockhart P, Guthrie B Trends in primary care antidepressant prescribing 1995–2007: a longitudinal population database analysis. Br J Gen Pract 2011; 61(590): e565–72. 22152736 10.3399/bjgp11X593848 PMC3162179

[CIT0011] Macri J C, Iaboni A, Kirkham J G, Maxwell C, Gill S S, Vasudev A, Whitehead M, Seitz D P Association between antidepressants and fall-related injuries among long-term care residents. Am J Geriatr Psychiatry 2017; 25(12): 1326–36. 28943234 10.1016/j.jagp.2017.08.014

[CIT0012] Maher R L, Hanlon J, Hajjar E R Clinical consequences of polypharmacy in elderly. Expert Opin Drug Saf 2014; 13(1): 57–65. 24073682 10.1517/14740338.2013.827660 PMC3864987

[CIT0013] Marengoni A, Onder G, Degli Esposti L, Russo P, Sangiorgi D, Buda S, Fini M, Marchionni N, Bonassi S, Mammarella F, Marrocco W, Pozzi G, Palmer K, Monaco A, Pecorelli S, Pani L Adherence to selective serotonin and serotonin-norepinephrine reuptake inhibitor prescriptions affects overall medication adherence in older persons: evidence from the Italian Nationwide OsMed Health-DB Database. J Clin Psychiatry 2016; 77(12): 1712–18. 28086009 10.4088/JCP.15m10503

[CIT0014] Mars B, Heron J, Kessler D, Davies N M, Martin R M, Thomas K H, Gunnell D Influences on antidepressant prescribing trends in the UK: 1995–2011. Soc Psychiatry Psychiatr Epidemiol 2017; 52(2): 193–200. 27885400 10.1007/s00127-016-1306-4 PMC5329088

[CIT0015] McCrea R L, Sammon C J, Nazareth I, Petersen I Initiation and duration of selective serotonin reuptake inhibitor prescribing over time: UK cohort study. Br J Psychiatry 2016; 209(5): 421–6. 27539294 10.1192/bjp.bp.115.166975

[CIT0016] Parker M, Johansen A Hip fracture. BMJ 2006; 333(7557): 27–30. 16809710 10.1136/bmj.333.7557.27 PMC1488757

[CIT0017] Pedersen A B, Mikkelsen E M, Cronin-Fenton D, Kristensen N R, Pham T M, Pedersen L, Petersen I Missing data and multiple imputation in clinical epidemiological research. Clin Epidemiol 2017; 9: 157–66. 28352203 10.2147/CLEP.S129785 PMC5358992

[CIT0018] Roche J J, Wenn R T, Sahota O, Moran C G Effect of comorbidities and postoperative complications on mortality after hip fracture in elderly people: prospective observational cohort study. BMJ 2005; 331(7529): 1374. 16299013 10.1136/bmj.38643.663843.55 PMC1309645

[CIT0019] Schmidt M, Pedersen L, Sorensen H T. The Danish Civil Registration System as a tool in epidemiology. Eur J Epidemiol 2014; 29(8): 541–9. 24965263 10.1007/s10654-014-9930-3

[CIT0020] Schmidt M, Schmidt S A, Sandegaard J L, Ehrenstein V, Pedersen L, Sorensen H T The Danish National Patient Registry: a review of content, data quality, and research potential. Clin Epidemiol 2015; 7: 449–90. 26604824 10.2147/CLEP.S91125 PMC4655913

[CIT0021] Schneeweiss S, Avorn J A review of uses of health care utilization databases for epidemiologic research on therapeutics. J Clin Epidemiol 2005; 58(4): 323–37. 15862718 10.1016/j.jclinepi.2004.10.012

[CIT0022] Souverein P C, Abbing-Karahagopian V, Martin E, Huerta C, Abajo F, Leufkens H G M, Candore G, Alvarez Y, Slattery J, Miret M, Requena G, Gil M J, Groenwold R H H, Reynolds R, Schlienger R G, Logie J W, Groot M C H, Klungel O H, Staa T P, Egberts T C G, De Bruin M L, Gardarsdottir H Understanding inconsistency in the results from observational pharmacoepidemiological studies: the case of antidepressant use and risk of hip/femur fractures. Pharmacoepidemiol Drug Saf 2016; 25(S1): 88–102. 27038355 10.1002/pds.3862

[CIT0023] Statistics Denmark Statistical Yearbook 2017 Copenhagen, Statistics Denmark; 2017.

[CIT0024] Sørensen H T, Christensen T, Schlosser H K, Pedersen L Use of Medical Databases in Clinical Epidemiology. Aarhus, SUN-TRYK, Aarhus Universitet; 2009.

[CIT0025] Topiwala A, Chouliaras L, Ebmeier K P Prescribing selective serotonin reuptake inhibitors in older age. Maturitas 2014; 77(2): 118–23. 24369815 10.1016/j.maturitas.2013.11.006

[CIT0026] Vieira E R, Palmer R C, Chaves P H Prevention of falls in older people living in the community. BMJ 2016; 353: i1419. 27125497 10.1136/bmj.i1419

[CIT0027] Zhou C, Fang L, Chen Y, Zhong J, Wang H, Xie P Effect of selective serotonin reuptake inhibitors on bone mineral density: a systematic review and meta-analysis. Osteoporos Int 2018; 29(6): 1243–51. 29435621 10.1007/s00198-018-4413-0

