# Supplementary material for: Selective serotonin reuptake inhibitor use in hip fracture patients: a Danish nationwide prevalence study
Source: Acta Orthop. 2018 Dec 10;90(1):33–9. doi: 10.1080/17453674.2018.1543842 (PMC6366466; doi:10.1080/17453674.2018.1543842)
Supplement: Supplemental Material [file IORT_A_1543842_SM4576.pdf]

Supplementary data

Table 1. International Classification of Diseases 10th revision (ICD-10) diagnosis codes from the Danish National Patient Registry

Disease	ICD-10 diagnosis codes
Myocardial infarction	I21; I22; I23
Congestive heart failure	I50; I11.0; I13.0; I13.2
Peripheral vascular disease	I70; I71; I72; I73; I74; I77
Cerebrovascular disease	I60–I69; G45; G46
Dementia	F00–F03; F05.1; G30
Chronic pulmonary disease	J40–J47; J60–J67; J68.4; J70.1; J70.3; J84.1; J92.0; J96.1; J98.2; J98.3
Connective tissue disease	M05; M06; M08; M09; M30; M31; M32; M33; M34; M35; M36; D86
Ulcer disease	K22.1; K25–K28
Liver disease	B18; K70.0–K70.3; K70.9; K71; K73; K74; K76.0; B15.0; B16.0; B16.2; B19.0; K70.4; K72; K76.6; I85
Diabetes type 1 and type 2	E10.0, E10.1; E10.9; E11.0; E11.1; E11.9; E10.2–E10.8; E11.2–E11.8
Hemiplegia	G81; G82
Moderate to severe renal disease	I12; I13; N00–N05; N07; N11; N14; N17–N19; Q61
Cancer	C00–C75; C91–C95; C81–C85; C88; C90; C96; C76–C80

Table 2. Codes from the Danish Multidisciplinary Hip Fracture Registry

Description	Codes
Housing	
Procedure code specifying housing	ZZ8050
Own accommodation	ZRSB01
Own accommodation in association with an institution	ZRSB01A
Homeless	ZRSB02
Residential institution	ZRSB04
No information on housing	ZRSB09
Body mass index	
Procedure code determining BMI	ZZ0242
BMI 10–80	VPH0010–VPH0080, VPK10K00–VPK80K00

Table 3. Anatomical therapeutic chemical (ATC) codes from the Danish National Database of Reimbursed Prescriptions

Medication	ATC codes
SSRI	
Citalopram	N06AB04
Escitalopram	N06AB10
Fluoxetine	N06AB03
Fluvoxamine	N06AB08
Paroxetine	N06AB05
Sertraline	N06AB06
Other medication	
Non-SSRI antidepressants	N06AA, N06AF, N06AG, N06AX
Antipsychotics	N05A
Antithrombotic medicine	B01A
Non-steroidal anti-inflammatory drugs	M01A
Corticosteroids	H02AB
Statins	C10AA

Table 5. Hip fracture patient characteristics according to selective serotonin reuptake inhibitor (SSRI) use 2006–2016. Values are frequency (%)

Variable	*Total*	*SSRI users*	*Non-users*
Total	*68,607 (100)*	*16,081 (23)*	*52,526 (77)*
Marital status			
Married	*20,341 (30)*	*4,205 (21)*	*16,136 (79)*
Unmarried	*48,266 (70)*	*11,876 (25)*	*36,390 (75)*
Housing			
Own accommodation	*31,076 (45)*	*5,538 (18)*	*25,538 (82)*
Homeless	*22 (< 1)*	*2 (9)*	*20 (91)*
Residential institution	*7,105 (10)*	*2,892 (41)*	*4,213 (59)*
Unknown	*30,404 (44)*	*7,649 (25)*	*22,755 (75)*
Body mass index			
< 18.5: Underweight	*6,003 (9)*	*1,433 (24)*	*4,570 (76)*
≥ 18.5 < 25: Normal weight	*31,635 (46)*	*7,206 (23)*	*24,429 (77)*
≥ 25: Overweight or obese	*17,640 (26)*	*4,023 (23)*	*13,617 (77)*
Unknown	*13,329 (19)*	*3,419 (26)*	*9,910 (74)*
Comorbidity			
Myocardial infarction	*3,769 (5)*	*943 (25)*	*2,826 (75)*
Congestive heart failure	*6,298 (9)*	*1,659 (26)*	*4,639 (74)*
Peripheral vascular disease	*5,522 (8)*	*1,454 (26)*	*4,068 (74)*
Cerebrovascular disease	*12,702 (19)*	*4,220 (33)*	*8,482 (67)*
Dementia	*6,747 (10)*	*2,883 (43)*	*3,864 (57)*
Chronic pulmonary disease	*8,674 (13)*	*2,476 (29)*	*6,198 (71)*
Connective tissue disease	*3,247 (5)*	*763 (23)*	*2,484 (77)*
Ulcer disease	*3,868 (6)*	*1,185 (31)*	*2,683 (69)*
Liver disease	*871 (1)*	*225 (26)*	*646 (74)*
Diabetes type 1 and 2	*6,705 (10)*	*1,690 (25)*	*5,015 (75)*
Hemiplegia	*175 (< 1)*	*64 (37)*	*111 (63)*
Moderate to severe renal disease	*2,694 (4)*	*718 (27)*	*1,976 (73)*
Cancer	*10,974 (16)*	*2,616 (24)*	*8,358 (76)*
